# Effects of family function, depression, and self-perceived burden on loneliness in patients with type 2 diabetes mellitus: a serial multiple mediation model

**DOI:** 10.1186/s12888-023-05122-y

**Published:** 2023-08-30

**Authors:** Yu Zhang, Xiangning Li, Yaxin Bi, Yinshi Kan, Hongyuan Liu, Lin Liu, Yan Zou, Ning Zhang, Li Fang, Weijuan Gong

**Affiliations:** 1 School of Nursing, Yangzhou University, 136 Jiangyang middle road, Yangzhou City, Jiangsu Province 225009 China; 2 Jiangsu Key Laboratory of Integrated Traditional Chinese and Western Medicine for Prevention and Treatment of Senile Diseases, Yangzhou, China; 3 Northern Jiangsu People’s Hospital, Yangzhou, 225009 China; 4 Affiliated Hospital of Yangzhou University, Yangzhou, 225009 China; 5 Department of Basic Medicine, School of Medicine, Yangzhou University, 136 Jiangyang Road, Yangzhou, Jiangsu 225009 China

**Keywords:** Type 2 diabetes mellitus, Loneliness, Family function, Depression, Self-perceived burden, Serial multiple mediation effect

## Abstract

**Background:**

Type 2 Diabetes mellitus (T2DM) has become a major lifestyle disease endangering human health worldwide. Patients with T2DM face varying degrees of loneliness, which adversely affects their family and the larger society. This study investigates the serial multiple mediating roles of depression and self-perceived burden between family function and loneliness in the T2DM population of China.

**Methods:**

In total, 260 T2DM patients were included. They rated themselves based on UCLA Loneliness Scale, Self-Rating Depression Scale, Self-Rating Anxiety Scale, Family Care Index, and Self-Perceived Burden Scale. Pearson and Spearman correlation analyses were conducted to clarify the association among variables. The SPSS macro-PROCESS program was used for a series of multiple mediation analyses.

**Results:**

Family function, depression, self-perceived burden, and loneliness were significantly correlated (*P* < 0.01). Family function not only has a direct negative impact (effect = -2.809; SE = 0.213; 95%CI: LL = -3.228, UL = -2.390) on loneliness, but also has an indirect impact on loneliness through the independent mediating role of depression (effect = -0.862; SE = 0.165; 95%CI: LL = -1.202, UL = -0.567) and self-perceived burden (effect = -0.288; SE = 0.107; 95%CI: LL = -0.525, UL = -0.114) and the chain mediating role of depression and self-perceived burden (effect = -0.202; SE = 0.066; 95%CI: LL = -0.342, UL = -0.088).

**Conclusions:**

Diversified interventions aimed at improving family function of T2DM patients would help in reducing the level of depression and self-perceived burden, and ultimately reducing loneliness.

## Introduction

Type 2 Diabetes mellitus (T2DM) is a lifelong lifestyle condition caused by the interaction among various genetic and environmental factors [1]. According to the 10th edition of the *IDF Diabetes Atlas*, approximately 537 million adults (20–79 years old) worldwide have diabetes in 2021. This figure is estimated to increase to 643 million and 783 million by 2030 and 2045, respectively [2]. China, the country with the largest number of diabetes patients, reported 156 million people with diabetes in 2020. Of these, 90–95% had T2DM [3].

T2DM not only endangers the physical health of patients, but also adversely affects their mental health. Some common psychological disorders that have been reported in T2DM patients include anxiety [4], depression [4, 5], diabetes-related distress [5–7], fear of hypoglycemia [6, 8], and loneliness, which has been reported recently [9]. Loneliness is a subjective psychological experience caused by the gap between an individual’s desire for communication and actual communication [10, 11]. Approximately one-fifth of Britons [12] and one-third of Americans [13] reported to have experienced loneliness.

At present, the research on loneliness mostly focuses on the elderly [14], adolescents [15], and other normal population. A small number of studies have focused on patients with cancer [16], schizophrenia [17], stroke [18], and other acute and critical conditions.Approximately 25–53% of T2DM patients have reported to suffer from moderate or above loneliness [19–21]. In severely lonely people with T2DM, the risk of death may increase by 22–26% compared to that in the normal population [22]. Therefore, it is highly important to pay attention to and attempt to reduce loneliness in T2DM patients in order to control their condition and improve their physical and mental health.

Family function, a concept that reflects the characteristics related to family relations, family intimacy, and adaptability, is being advocated as a protective factor against loneliness [23–25]. However, the current research on the association of loneliness with family function mostly focuses on the normal populationand no direct study has been conducted on the relationship between the above two factors in T2DM population. Despite this, some studies have reported that good family function can help TDM patients in regulating their blood glucose fluctuations, strengthening their psychological elasticity, and inhibiting the development of negative emotions such as anxiety and depression [26]. These studies reasonably predicts that a correlation exists between family function and loneliness in T2DM patients.

However, the mechanism through which family function affects loneliness in T2DM patients need to be further studied. Existing studies have shown that depression induces loneliness [27–29]. Good family function can effectively reduce the occurrence or level of depression in postpartum women [30, 31], the elderly [32], and epileptic children [33]. Some studies have also reported that in T2DM patients, good family function can enhance the psychological elasticity of patients, inhibit the generation of negative emotions such as anxiety and depression symptoms, and help regulate the blood glucose fluctuations [26]. Considering the above relationship among family function, depression, and loneliness, we herein aim to verify whether depression has a mediating effect between family function and loneliness in the T2DM population.

Self-perceived burden refers to the patient’s guilt of using the help of a caregiver for daily life activities and the resulting frustration about oneself [34, 35]. A small number of studies have shown that self-perceived burden can affect loneliness [27, 36]. Self-perceived burden is common in T2DM patients. Yu et al. [37] investigated 215 patients with diabetes and observed that the self-perceived burden of T2DM patients was at a mild-to-moderate level. A study on patients with diabetic foot showed that 88% of the patients had different degrees of self-perceived burden; the higher the severity of the disease, the heavier the self-perceived burden [38–42]. Although not confirmed in the T2DM population, depressive symptoms are a significant predictor of self-perceived burden in patients with chronic pain [43]. Good family function can significantly reduce the level of self-perceived burden in patients suffering from maintenance hemodialysis [44] and post-breast cancer surgery [45] and in elderly patients with coronary stent implantation [46]. To sum up, we hypothesized that in T2DM population, depression may affect self-perceived burden, which may be a potential mediator between family function and loneliness.

From the above discussion, we can see that the family function, depression, self-perceived burden, and loneliness might be related. The current study examines the mediating effects of depression and self-perceived burden between family function and loneliness in the T2DM population of China. For this purpose, we propound the following assumptions. Firstly, there is a possible correlation between family function and loneliness in the T2DM patients of China. Secondly, depression may mediate the relationship between family function and loneliness. Then, self-perceived burden may mediate this relation between family function and loneliness. Finally, “chain” mediating effect on depression and self-perceived burden together on the relationship between family function and loneliness.

## Methods and measurements

### Data source and sample

Convenience sampling was adopted to recruit patients with T2DM. All the participants were recruited from two tertiary hospitals (Yangzhou city, Jiangsu Province, China) from February 2021 to June 2021. The following inclusion criteria were used: (1) those diagnosed with T2DM for at least 1 year; (2) ≥ 18 years of age; (3) had good communication and verbal skills; and (4) were willing to participate in the study. The following exclusion criteria were used: (1) patients with acute complications; (2) those with limited vision because of complications or comorbidities; (3) those with severe comorbid psychiatric disorders; or (4) those who did not have the ability to read and write in Chinese. The survey included demographic characteristics, family function, depression, self-perceived burden, and loneliness.

According to the Kendall sample estimation method, the sample size for multivariate analysis was 5–10 times the variables of the study. Considering the maximum multiple and 15% invalid questionnaire, our sample size can be calculated as follows: number of independent variables×10 × (1 + 15%). A total of 275 questionnaires were distributed among the participants, and 260 valid questionnaires were recovered, with a valid recovery rate of 94.50%, which met the survey requirements. Written informed consent was obtained from patients, who were then instructed to complete the questionnaire independently and anonymously. This study was approved by the ethics committee of Yangzhou University (YZUHL20210087), China.

### Measurement tools

#### General information questionnaire

The data were collected using a self-designed questionnaire. The following information were collected: age, sex, diabetes symptoms, duration of diabetes, family history, complications (retinopathy, neuropathy, nephropathy, foot ulcers, and cardiovascular complications), marital status, residence status, and the most recent glycated hemoglobin (HbA1c) levels.

#### Family function

The Family APGAR scale, designed by Smilkstein, was used to assess the family function. It includes five items: adaptation, partnership, growth, affection, and resolution [47]. Each item is scored on a 3-point Likert scale: 0 (almost never), 1 (some of the time), and 2 (almost always). The total score can have values ranging from 0 to 10. The higher the score, the better the family function. The total score is divided into three levels from 0 to 10: 0–3 indicates severe family dysfunction, 4–6 indicates moderate family dysfunction, and 7–10 indicates good family function. The APGAR scale is widely used as it has good reliability and validity. In this study, the Cronbach’s alpha coefficient for the family function was 0.853.

#### Depression

The Self-Rating Depression Scale (SDS) was developed by Zung in 1965. It is used to evaluate the severity of the depressive state of study participants during the past week [48]. The SDS consists of 20 items, each of which is scored on a four-point Likert scale (1, no or seldom; 2, sometimes; 3, most of the time; 4, most or all of the time). Of these 20 items, 10 express negative experiences or symptoms and are scored positively, while the other 10 express positive experiences and are scored negatively. The total score is calculated by adding the initial score of the 20 items and multiplying them by 1.25. Patients are classified according to their total score on the SDS as follows: normal (total score: <50), mild depression (total score: between 50 and 59), moderate depression (total score: between 60 and 69), and severe depression (total score: ≥70). The Cronbach’s alpha coefficient for the SDS was 0.862 [49].

#### Self-perceived burden

The Self-Perceived Burden Scale (SPBS), developed by Cousineau et al. [34], consists of 10 items covering three dimensions: body burden, economic burden, and emotional burden. The SPBS score uses a five-point Likert scale, from “never” (1 point) to “always” (5 points). The total score is the sum of individual items (only the eighth item was reverse scored; all the others were positive). The SPBS score is classified into the following four groups: <20, not significant; 20–29, mild; 30–39, moderate; and ≥ 40, severe self‐perceived burden. The higher the total score, the higher the individual’s SPB level. In this study, the Cronbach’s alpha coefficient for SBP was 0.844.

#### Loneliness

The UCLA Loneliness Scale (Version 3), developed by Russel et al. [50], is used to assess participants’ level of loneliness. The scale consists of 20 items on a four-point scale ranging from “never felt this way” to “always felt this way,” with a total score of 20–80. The higher the score is, the higher the loneliness degree is. The Cronbach’s alpha coefficient for the UCLA was 0.887 [51].

### Statistical analysis

The SPSS 26.0 software was used for data analysis. Measurements that conform to a normal distribution were expressed as the mean ± standard deviation, and those that do not were expressed as the median and quartile spacing, M (QR). Pearson correlation analysis was used for variables conforming to normal distribution, while Spearman rank correlation analysis was used for those not conforming to normal distribution. *P* < 0.05 indicated statistical difference. Process 3.3 was used to analyze the mediating effect of the data. The Bootstrap sample number was set to 2000, and a 95% confidence interval was set. If the upper and lower limits did not include 0, it was statistically significant.

## Results

### Characteristics of samples

The socio-demographic characteristics of the sample are displayed in Table 1. A total of 260 patients with T2DM were recruited for this study, including 145 men (55.8%) and 115 women (44.2%), with an average age of (59.35 ± 14.20) years. The married population accounted for 90.0% of the total population. The majority of participants had personal monthly incomes in the ¥2000–4999 range (*n* = 156, 60.0%). Among the patients, 145 (55.8%) had a ≤ 10 years history of diabetes, while 115 (44.2%) had a > 10 years history. In addition, 63.5% of the patients had comorbidities.

**Table 1:** The characteristics of samples (N = 260)

Variables	Category	Number	Percentage (%)
**Gender**			
	Male	145	55.8
	Female	115	44.2
**Age**			
	18 ~ 44	38	14.6
	45 ~ 59	81	31.2
	≥ 60	141	54.2
**Marriage**			
	Married	234	90.0
	Unmarried	12	4.6
	Divorced or widowed	14	5.4
**Income(Yuan/month)**			
	0 ~ 1999	19	7.3
	2000 ~ 4999	156	60.0
	5000 ~ 9999	62	23.9
	≥ 10,000	23	8.8
**Duration of diabetes(year)**			
	≤ 10	145	55.8
	>10	115	44.2
**Family History**			
	Yes	73	28.1
	No	187	71.9
**Complications**			
	0	95	36.5
	1 ~ 2	117	45.0
	3 ~ 6	39	15.0
	≥ 7	9	3.5
**BMI**			
	<18.5	6	2.3
	18.5 ~ 23.9	109	41.9
	≥ 24	145	55.8
**Smoking**			
	Yes	95	36.5
	No	165	63.5
**Drinking**			
	Yes	84	32.3
	No	176	67.7
**Family Environment**			
	Good	115	44.2
	General	135	51.9
	Bad	10	3.9
**Fasting blood glucose**			
**(mmol/L)**	3.9 ~ 6.1	45	17.3
	>6.1	215	82.7
**HbA1c(%)**			
	<7	25	9.6
	≥ 7	235	90.4

Note: BMI, Body mass index;HbA1c, Hemoglobin A1C.

### Descriptive statistics for loneliness, depression, self-perceived burden, and family function

**Fig. 1:**
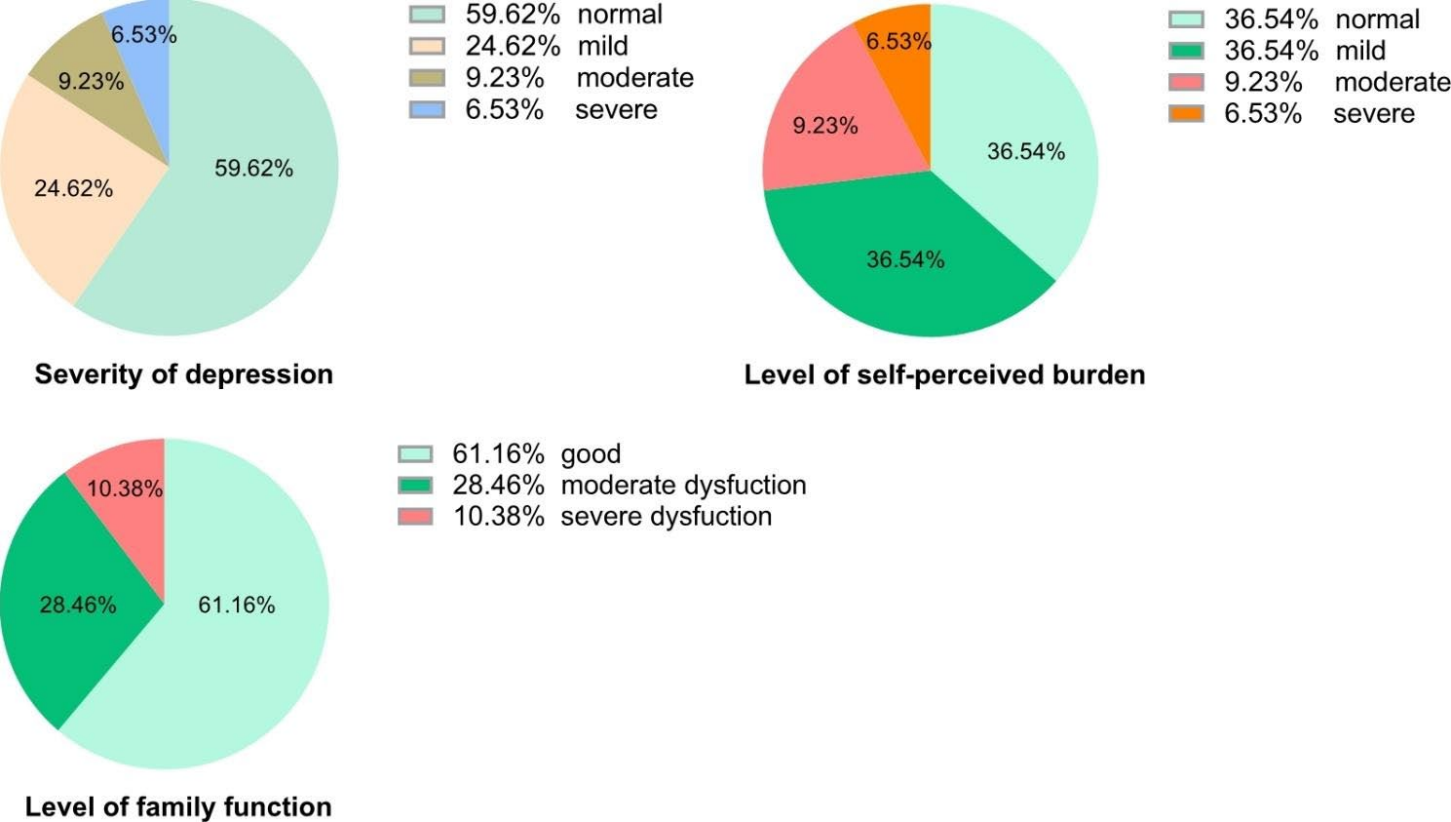
Descriptive statistics for scales

Table 2; Fig. 1 show the score of different scales. The average UCLA loneliness scale score was 41.76 ± 11.75, with a minimum of 20 points and a maximum of 74 points. The average SDS score was 50.39 ± 11.92. It was ≥ 50 for 105 (40.3%) patients, who were classified as having depressive symptoms. The average score of SPBS was 22.93 ± 9.36, and the overall average was at the level of mild self-perceived burden. The APGAR mean score was 7.20 ± 2.65.

**Table 2:** Descriptive statistics for variables

Variable(score)	Minimum	Maximum	Mean (SD)
UCLA-LS	20	74	41.76 (11.75)
SDS	29	81	50.39 (11.92)
SPBS	10	50	22.93 (9.36)
APGAR	0	10	7.20 (2.65)

Note: SD, Standard Deviation

### Correlation of depression, self-perceived burden, family function and loneliness

Table 3 lists the correlations among research variables with statistical differences of all analysis results (*P* < 0.01 (two-tailed)). Firstly, a significant negative relationship was noted between family function and loneliness, with a correlation coefficient of -0.609. Secondly, depression and self-perceived burden were positively correlated with loneliness (*r* = 0.642 and *r* = 0.588, respectively). Depression is also positively correlation with self-perceived burden (*r* = 0.574). Finally, family function was significantly negatively correlated with both depression and self-perceived burden (*r* = -0.467 and *r* = -0.543, respectively).

**Table 3:** Correlations between depression, self-perceived burden, family function and loneliness

	Depression	Self-perceived burden	Family function	Loneliness
Depression	1			
Self-perceived burden	0.574**	1		
Family function	-0.467**	-0.543**	1	
Loneliness	0.642**	0.588**	-0.609**	1

Note: **P < 0.01 (two-tailed)

Loneliness and depression, self-perceived burden, family function were for linear regression analyses (Table 4). Model 1 shows that loneliness is associated with Depression (β = 0.371, *P* < 0.001), self-perceived burden (β = 0.201, *P* < 0.001) and family function (β= -0.329, *P* < 0.001). Model 2, after adjusting for age and gender, shows that depression, self-perceived burden, family function are associated with loneliness (depression: β = 0.370, *P* < 0.001; self-perceived burden: β = 0.209, *P* < 0.001; family function: β= -0.328, *P* < 0.001). Model 3, after adjusting for age, gender, marriage, income and duration of diabetes, showed loneliness were still associated with depression, self-perceived burden and family function (depression: β = 0.351, *P* < 0.001; self-perceived burden: β = 0.200, *P* < 0.001; family function: β= -0.272, *P* < 0.001).

**Table 4:** Linear regression analyses between loneliness and depression, self-perceived burden, family function

	Model 1			Model 2			Model 3		
	β	P	95%CI	β	P	95%CI	β	P	95%CI
Depression	0.371	< 0.001	0.262 to 0.469	0.370	< 0.001	0.260 to 0.420	0.351	< 0.001	0.245 to 0.446
Self-perceived burden	0.201	< 0.001	0.118 to 0.386	0.209	< 0.001	0.127 to 0.398	0.200	< 0.001	0.116 to 0.385
Family function	-0.329	< 0.001	-1.900 to -1.013	-0.328	< 0.001	-1.897 to -1.009	-0.272	< 0.001	-1.639 to -0.766

Note: Model 1: Unadjusted; Model 2: Adjusted age and gender; Model 3: Adjusted age, gender, marriage, income, duration of diabetes. Abbreviations: β, regression coefficient; 95% confidence interval

### Mediation analysis of depression and self-perceived burden between family function and loneliness

**Fig. 2:**
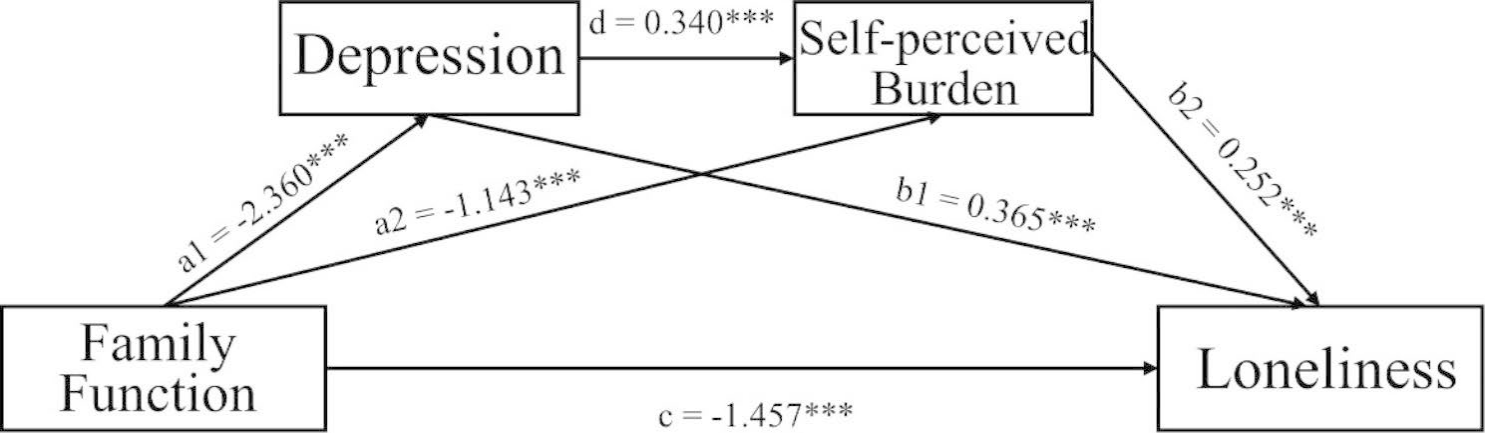
Serial mediation models for family function, depression, self-perceived burden and loneliness Note: Path coefficients were shown in standardized regression coefficient. *** *P*<0.001

The results of relationship between family function and loneliness are shown in Table 5; Fig. 2, respectively. Firstly, family function exerts a negatively significant influence on loneliness in T2DM patients (*P* < 0.001); the higher the level of family function was, the lower the level of loneliness was. In addition, all the three indirect paths were also significant. The first indirect way was that the effect of family function on loneliness was independently mediated by depression, with an effect value of 0.862. The second indirect way was that self-perceived burden significantly mediated the effect of family function on loneliness, with an affect value of 0.288. Finally, the indirect effect of family function on loneliness was also found to be significant through the chain mediating role of depression and self- perceived burden, and its effect value was 0.202. The 95% confidence intervals (CI) of these three indirect paths did not contain zero, indicating that the mediating effect of the three paths was significant.

**Table 5:** Hypothesized serial mediation model of depression and self-perceived burden between family function and loneliness

Pathway	β(SE)	95%CI
**Total effect**	-2.809 (0.213)	-3.228 to -2.390
**Direct effect**	-1.457 (0.225)	-1.901 to -1.013
Fun → Dep	-2.360 (0.237)	-2.828 to -1.892
Fun → Bur	-1.143 (0.193)	-1.523 to -0.762
Dep → Lon	0.365 (0.052)	0.262 to 0.469
Bur → Lon	0.252 (0.068)	0.118 to 0.387
Dep → Bur	0.340 (0.043)	0.256 to 0.425
**Indirect effects**		
Total indirect effect	-1.353 (0.190)	-1.742 to -1.005
Indirect 1	-0.862 (0.165)	-1.202 to -0.567
Indirect 2	-0.288 (0.107)	-0.525 to -0.114
Indirect 3	-0.202 (0.066)	-0.342 to -0.088

Note: SE, Standard error; β,regression coefficient; 95% CI, 95% confidence interval;Fun, Family function; Dep, Depression; Bur, Self-perceived burden; Lon, Loneliness; indirect 1, family function → depression → loneliness; indirect 2, family function → self-perceived burden → loneliness; indirect 3, family function → depression → self-perceived burden → loneliness

## Discussion

We mainly tested whether depression and self-perceived burden regulated the relevance between family function and loneliness in T2DM patients. The results showed that, firstly, family function could directly and significantly affect the loneliness in T2DM patients. Secondly, depression and self-perceived burden partially mediated family function and loneliness. Thirdly, depression and self-perceived burden have a chain mediating effect. Hence, we were able to confirm the hypothesized relationships that we proposed for family function, depression, self-perceived burden, and loneliness.

### Direct influence of family function on loneliness

Our study results showed that family function can directly affect loneliness in T2DM patients; the higher the level of family function, the lower the loneliness patients felt. The degree of time and space shared between patients and their family members best determines the level of loneliness in patients. Family function is a direct predict factor of loneliness. Disorganized family management or lack of intimate contact between family members causes loneliness among patients [52]. Some studies have also shown that family function is significantly negatively correlated with social loneliness in the normal population, with higher levels of family closeness and adjustment being associated with greater affection and love and lesser social isolation [53]. Patients with T2DM tend to have a higher dependence on their families. Therefore, it is necessary for them to have good family function to help them build an effective psychological barrier to protect them against external stimulus sources that cause mood swings and negative emotions. Therefore, the role of family function in causing loneliness in T2DM patients should not be ignored. Patients should be encouraged to actively express their needs and emotional experiences to their family members, who, in turn, should be advised to provide emotional comfort and timely support to patients to avoid and/or reduce their loneliness [54].

### Mediation effect of depression and self-perceived burden

We also explored the underlying mechanisms of family function and loneliness in patients with T2DM. This study demonstrates that the indirect association between family function and loneliness can be independently mediated by depression or self-perceived burden in T2DM patients.

This study showed that depression plays a mediating role between family function and loneliness. The relation between depression and loneliness has been well documented [55–57]. Cacioppo et al. [58] demonstrated a strong association between depression and loneliness in older adults. In addition, depressive symptoms and loneliness can cooperate with each other to reduce the happiness degree of the elderly. A longitudinal study lasting five years in older adults of Chicago showed that loneliness indicates a subsequent increase in depression [59]. An increase in depressive symptoms can predict loneliness [60]. As most patients with T2DM choose home for follow-up treatments, family dysfunction may alienate them and their family members. The probability of negative emotions such as depression and anxiety greatly increased in patients in a long-term, closed, and depressing living environment [61]. These negative emotions can cause patients to avoid social interaction, which reduces their social skills over time and further decreases the frequency or quality of their social activities, thereby increasing loneliness [62]. Long-term negative psychology can break down the psychological defenses of a patient. Our study demonstrated that depression independently mediates the influence of family function on loneliness in T2DM population. Therefore, diversified interventions should be used to improve the level of family function or reduce the depression, so as to reduce loneliness.

This study showed that self-perceived burden mediates the influence of family function on loneliness in T2DM population. We also showed that good family function reduces the level of self-perceived burden. Similar results were reported by Kuo et al. [63], who investigated the role of family in cancer patients. Moreover, Li et al. [64], using a model of care, demonstrated that the family members of lung cancer patients had a lower self-perceived burden. In addition, McPherson et al. [65] found that care from a partner is closely related to the self-perceived burden of stroke patients and may affect the quality of life of patients. Hence, family function plays an important role in influencing the self-perceived burden in T2DM patients. Support received from family members might reduce patients’ stress, improve their confidence, and reduce the self-perceived burden. Our results also suggest that self-perceived burden can impact loneliness. Ejerskov et al. [27] and Hill and Frost [36] reported that self-perceived burden could indirectly affect the patients’ feelings of loneliness. Our findings revealed the independent mediating role of self-perceived burden in the association of family function and loneliness in T2DM patients, which suggests that targeted measures to improve family function or reduce self-perceived burden can be effective in reducing levels of loneliness.

### Chain mediating effect of depression and self-perceived burden in the relationship between family function and loneliness

In addition to examining the independent mediating role of depression and self-perceived burden, our study also tested a potential chain mediating role between family function and loneliness. Our findings suggest the accumulative mediating role of depression and self-perceived burden in the family function and loneliness. Many previous studies have shown that good family function can help improve the mental health of patients with T2DM [30, 31]. Good family function can enhance the psychological elasticity of patients and inhibit the generation of negative emotions, and hence, reduce their depression. In addition, the reduction in depression also helps in decreasing the self-perceived burden in patients with diabetes [66]. Finally, self-perceived burden is positively related to loneliness [36]. Patients who had a higher level of self-perceived burden tended to have greater loneliness. Our findings suggested that more attention should be paid to T2DM patients with loneliness. Interventions aimed at strengthening the family function and reducing the level of depression and self-perceived burden should be adopted by medical personnel.

## Limitations

Our study has a few shortcomings. First, the participants were taken from only two large general hospitals in China. This group may not be sufficient to represent the larger patient population with T2DM, which limits the generalizability of the findings. In addition, the cross-sectional study cannot explain the causal relationship between the different variables. Hence, future longitudinal studies need to be designed to further explore the dynamic effects of depression, self-perceived burden, and family function on loneliness.

## Conclusion

Family function can not only have a direct negative impact on loneliness in patients with T2DM, but also have an indirect impact on loneliness through the independent mediating role of depression and self-perceived burden and the chain mediating role of depression and self-perceived burden. Diversified interventions aimed at improving family function of T2DM patients would help in reducing the level of depression and self-perceived burden, and ultimately reducing loneliness.

## Data Availability

Data and materials used in this study are available from the corresponding authors and will be made available on reasonable request.

## Abbreviations

HbA1c: Hemoglobin
T2DM: Diabetes mellitus type 2
SDS: Self-Rating Depression Scale
SPBS: Self-Perceived Burden Scale
